# KAN-ResNet-Enhanced Radio Frequency Fingerprint Identification with Zero-Forcing Equalization

**DOI:** 10.3390/s25072222

**Published:** 2025-04-01

**Authors:** Hongbo Chen, Ruohua Zhou, Qingsheng Yuan, Ziye Guo, Wei Fu

**Affiliations:** 1 School of Electrical and Information Engineering, Beijing University of Civil Engineering and Architecture, Beijing 102616, China; 2 National Computer Network Emergency Response Technical Team Coordination Center of China, Beijing 100029, China

**Keywords:** radio frequency fingerprint identification, Internet of Things security, Wi-Fi, zero-forcing equalization, KAN-ResNet

## Abstract

Radio Frequency Fingerprint Identification (RFFI) is a promising device authentication technique that utilizes inherent hardware flaws in transmitters to achieve device identification, thus effectively maintaining the security of the Internet of Things (IoT). However, time-varying channels degrade accuracy due to factors like device aging and environmental changes. To address this, we propose an RFFI method integrating Zero-Forcing (ZF) equalization and KAN-ResNet. Firstly, the Wi-Fi preamble signals under the IEEE 802.11 standard are Zero-Forcing equalized, so as to effectively reduce the interference of time-varying channels on RFFI. We then design a novel residual network, KAN-ResNet, which adds a KAN module on top of the traditional fully connected layer. The module combines the B-spline basis function and the traditional activation function Sigmoid Linear Unit (SiLU) to realize the nonlinear mapping of the complex function, which enhance the classification ability of the network for RFF features. In addition, to improve the generalization of the model, the grid of B-splines is dynamically updated and L1 regularization is introduced. Experiments show that on datasets collected 20 days apart, our method achieves 99.4% accuracy, reducing the error rate from 6.3% to 0.6%, outperforming existing models.

## 1. Introduction

Radio Frequency Fingerprint Identification (RFFI) technology differentiates devices based on the hardware characteristics of radio frequency signals [1,2,3], serving as a lightweight, non-encryption-based access authentication (see Appendix A for definition) technique. It is widely used in the Internet of Things (IoT) domain, including applications such as smart healthcare [4], smart cities [5], and smart homes [6]. Traditional authentication schemes based on software addresses, such as Media Access Control (MAC) addresses, rely on credentials and encryption to grant network access. However, this approach has several inherent limitations [7]. The absence or limited use of encryption creates security vulnerabilities, exposing individual IoT devices and their infrastructure to potential exploitation by malicious attackers [8,9]. This concern is particularly critical for IoT devices, as their computational constraints limit the feasibility of strong encryption algorithms. Moreover, these authentication methods are prone to spoofing attacks [10,11], further weakening network security. As a result, the lack of strong encryption mechanisms poses a significant threat to cybersecurity [12], industrial robotic systems security [13], and IoT security. Radio Frequency Fingerprints (RFFs) originate from hardware imperfections introduced during the manufacturing process [14], and these hardware variations impart unique and inherent characteristics to transmitters, even within the same model or production batch [15]. These properties, acting on radio signals, create unintended modulation that, while not affecting regular communication or detection functions, slightly distorts the waveform, allowing hardware defects to be extracted as a unique device identifier. Similar to biological fingerprints, an RFF is unique and difficult to tamper with, significantly enhancing device resistance to spoofing attacks. Importantly, RFFI does not increase the power consumption of the authenticated devices [5,16], a critical advantage for IoT terminal nodes with limited computational and energy resources. Furthermore, various wireless signals, including Wi-Fi, GSM, CDMA, LTE, Bluetooth, ZigBee, LoRa [17], and RFID, can be used to extract RFFs, enabling precise device identification (see Appendix A for definition).

RFFI for wireless devices uses two main types of techniques: expert feature extraction and deep learning. Expert feature extraction relies on prior knowledge and typically uses techniques such as short-time Fourier transform, Wavelet transform, and constellation diagrams to convert I/Q data into time-frequency spectrograms [18], bispectral feature maps [19], constellation diagrams [20], and other feature representations. The advantage of this method lies in its ability to leverage domain expertise to maximize individual differences for specific problems. However, it also has several drawbacks. It relies on specialized knowledge and requires careful parameter selection for specific issues. Converting I/Q data into expert features increases the computational burden. Additionally, expert features may be tightly coupled with specific protocols. In contrast, deep learning methods can automatically extract high-level features directly from raw RF signals through hidden layers. When trained on large amounts of labeled data, deep neural networks can efficiently extract RFF features. Compared to expert feature methods, deep learning demonstrates superior performance in both recognition accuracy and efficiency.

The goal of deep learning in RFFI is to automatically detect subtle, imperceptible flaws in radio frequency signals without relying on extensive signal processing expertise, enabling end-to-end learning. Moreover, there is no need to pre-define the modulation format of the transmitted signal, making the extracted RFF features more difficult to forge. Common models include Convolutional Neural Networks (CNNs) [2,21,22,23,24,25], Residual Networks (ResNets) [26,27,28,29,30,31], Long Short-Term Memory networks (LSTMs) [32], Multilayer Perceptrons (MLPs) [33], and Transformers [34], etc. Among these, CNNs have been the earliest and most widely used network structure in RFFI due to their advantages of local receptive fields and weight sharing. Riyaz et al. [2] designed the RFF-CNN1 model, which consists of two convolutional layers and one fully connected layer. Restuccia et al. [21] developed a deeper model, DeepRadioID, by increasing the number of convolutional layers. Gopalakrishnan et al. [22] proposed the RFF-CNN2 model, whose basic unit consists of 16 1 × 4 convolutional layers, batch normalization layers, and max-pooling layers. Since CNN models may encounter issues such as network degradation and vanishing gradients, researchers have increasingly explored the application of residual networks in RFFI. Gritsenko et al. [26] designed the ResNet-50-1D model based on ResNet-50, while Zhang et al. [27] developed a smaller-parameter RFFResNet model. The residual design is a plug-and-play, simple yet effective method that can be easily integrated with other approaches. As the network depth increases, the model’s learning capacity improves, making it more suitable for handling large datasets.

Both expert feature extraction and deep learning methods experience a decline in RFFI accuracy when impacted by time-varying channels. This happens because expert feature extraction assumes that Wi-Fi device hardware imperfections stay relatively stable over time. However, RFF features are susceptible to interference from time-varying channels, resulting in decreased stability of feature recognition. Deep learning models often extract radio frequency features from training samples that include channel characteristics, which may differ in testing samples. When the channel conditions in testing samples differ from those in training samples, deep learning models experience a significant drop in recognition performance, reducing their generalization ability [28]. Tekbas et al. [35] were among the first to study the effects of environmental temperature, battery voltage, and ambient noise on transmitter classification success rates. Rehman et al. [36] further explored the influence of device aging, environmental temperature, and device mobility on RFF recognition. Gu et al. [37] investigated the effect of temperature on the stability of mobile phone radio frequency features and classified signals by manually selecting suitable RF features. He et al. [38] studied the impact of multipath fading channels on RFF recognition and proposed a channel-independent RFFI system based on spectral quotient constellation error, achieving high recognition accuracy. In practical applications, the signals collected during training are typically not from the same day as those used for recognition. Fadul et al. [39]. used a Conditional Generative Adversarial Network (CGAN) and the Joint Convolutional Auto-Encoder and Convolutional Neural Network (JCAE-CNN) to solve the problem of extracting RFF features under multipath fading conditions. Hanna et al. [23] demonstrated through experiments that when using a CNN to classify the same transmitter over time, accuracy gradually declines. However, by employing minimum mean square error (MMSE) equalization, recognition accuracy on the test set improved from 49.7% to 93.7%.

To address the above issues, this study proposes an RFFI method that combines Zero-Forcing (ZF) equalization with the KAN-ResNet model. First, we introduce ZF equalization during the data preprocessing stage. This method applies the pseudoinverse of the channel matrix to invert the received signal. Its core principle is to construct a matrix that makes the received signal as close as possible to the original signal. Inspired by [40], we design an innovative residual network structure, KAN-ResNet. Instead of traditional fully connected layers, it integrates KAN modules (Kolmogorov–Arnold Network modules) to extract and classify RFF features. After the residual network extracts the RFF features, we perform classification using the KANLinear layer. By incorporating B-spline basis functions and the SiLU activation function, the KANLinear layer enables nonlinear mapping of complex functions. Additionally, through dynamic grid updates and regularization loss, dynamic grid updates and regularization loss further enhance the generalization ability and recognition accuracy of the KAN-ResNet model. Notably, by replacing traditional fully connected layers with KANLinear layers, the number of network parameters is reduced. In summary, our goal is to solve two key challenges in RFFI: (1) removing the impact of time-varying channels on raw I/Q signals; (2) designing a deep learning model that does not rely on expert features, enabling end-to-end RFF identification.

The main contributions of this paper are summarized as follows:

1. We introduce zero-forcing equalization, a simple and efficient pre-processing method that completely reduces inter-symbol interference (ISI) due to multipath effects;
2. We designed a residual network based on a KAN. We proposed the KAN-Resnet model, which replaces the single complex activation function with a combination of multiple simple functions that are learnable, improving the performance and interpretability of the network;
3. We proposed a novel framework for RFFI. Through extensive experiments, this paper establishes an optimal RFFI framework by combining Zero-Forcing equalization with the KAN-ResNet model. When tested on transmitter signals collected 22 days apart, the accuracy reaches as high as 99.4%. Additionally, the model demonstrates the shortest training time (1 min 44 s) and the strongest robustness.

The remainder of this paper is organized as follows: Section 2 reviews related work. Section 3 describes the factors affecting RF signal generation and transmission. Section 4 presents the system structure of our method, and describes the data preprocessing steps for raw Wi-Fi signals, including the proposed ZF equalization. Section 5 details the proposed KAN-ResNet model. Section 6 provides the dataset, experimental setup, results, and performance comparisons of models. Finally, Section 7 summarizes the main contributions of the paper and outlines directions for future work.

## 2. Related Work

Deep learning-based RFFI methods typically consist of two steps: data processing and network classification. As the first step in RFFI, data processing aims to highlight key features and reduce interference, allowing more discriminative features to be extracted in subsequent steps. Restuccia et al. [21] and Al-Shawabka et al. [28] studied the impact of time-varying channels on RFFs. They applied time-domain equalization using Finite Impulse Response (FIR) filters to reduce wireless channel effects and improve RFFI. Sankhe et al. [41] removed channel effects from raw I/Q samples using down-conversion techniques. Fu et al. [42] proposed two channel equalization methods: time-domain Least Mean Square (LMS) equalization and classic Frequency-Domain Equalization (FDE), and designed a hybrid classifier combining the strengths of both methods to increase recognition accuracy. Hanna et al. [23] used MMSE equalization to mitigate the effects of time-varying channels on RFFI. Although these methods have shown promising performance in addressing channel effects, they still present several limitations. FIR requires per-device optimization, which increases implementation complexity and computational cost. LMS suffers from slow convergence and high computational complexity. FDE is highly sensitive to channel variations, resulting in the loss of certain RFF features. MMSE exhibits high computational complexity and relies on precise channel and noise information. In contrast, this paper proposes using ZF equalization to compensate for the impact of time-varying channels on raw I/Q signals. The ZF equalization algorithm is simpler and has lower computational complexity compared to the aforementioned methods.

In deep learning-based RFFI methods, CNNs are the most widely used models. However, as the network depth increases, CNNs are prone to issues such as gradient explosion and degradation. Al-Shawabka et al. [28] compared three network architectures and found that ResNet-50-1D performed best on the DARPA dataset. Their experiments showed that choosing the right deep learning model can greatly improve RFFI accuracy. Jian et al. [29] demonstrated that residual networks perform better in large-scale classification tasks, with higher overall performance. Zeng et al. [30] achieved good results by fusing multiple features and using a residual network for identification. Currently, most residual network models for RFFI still use fully connected layers to classify extracted features. Liu et al. [40], based on the Kolmogorov–Arnold theorem, designed a KAN network characterized by learnable activation functions, where each weight parameter is replaced by a learnable univariate function and parameterized in the form of spline functions. Cheon et al. [43] combined KAN networks with various CNNs for remote sensing classification tasks, showing that the KAN network achieved the same accuracy as MLP with fewer training epochs and parameters. Kocning et al. [44] integrated the KAN network with Neural Ordinary Differential Equations (Neural ODEs) to learn dynamic systems and hidden physical laws, achieving significant results with a smaller network architecture compared to traditional Neural ODEs. Tang et al. [45] improved the U-Net structure by combining it with a KAN network, finding that U-KAN achieved superior performance with fewer training epochs compared to U-Net. The objective of this paper is to design a new residual convolution model based on KAN and to prove the effectiveness of KAN in the field of RFFI, as well as to enhance the performance of deep learning in RFF feature extraction and identification. In the following sections, we will validate the proposed model through extensive experiments.

## 3. Problem Statement

**Figure 1:**
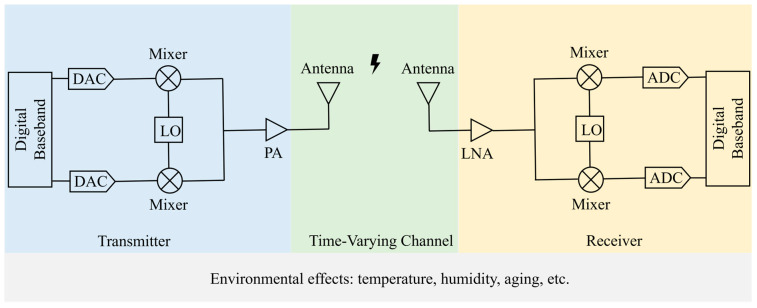
Factors affecting RFF generation, propagation, and reception. The diagram illustrates the various factors influencing the RFF during its generation, propagation, and reception. Key components include the Digital-to-Analog Converter (DAC), Local Oscillator (LO), Power Amplifier (PA), Low Noise Amplifier (LNA), and Analog-to-Digital Converter (ADC).

During the generation of Wi-Fi signals, hardware imperfections such as local oscillator frequency offset, phase noise, DAC distortions, and power amplifier nonlinearity affect the signal, collectively forming the unique RFF. Similarly, during signal propagation, wireless channel effects like antenna polarization, multipath fading, noise, and distortion can also impact the stability of the RFF. Upon signal reception, local oscillator frequency offset, phase noise, ADC distortions, and power amplifier nonlinearity at the receiver can further affect the RFF of the transmitter. In this study, we focus solely on RFF identification using data received by the same receiver, thus the effects of receiver variations can be ignored. All these factors are influenced by environmental conditions such as temperature, humidity, and device aging, as illustrated in Figure 1.

The hardware variations of the radiating source, including manufacturing tolerances and assembly differences in components such as the DAC, I/Q modulator, filters, and power amplifiers, affect the radio signal. The ultimately transmitted radio signal is:

(1) $$T_{x}\left(t\right)\;=\;\left[\;h_{tx}\;\frac{1}{2\pi }\;*\;\int_{-W/2}^{W/2}X\left(w\right)\;e^{jwt}\;dw\;\right]e^{jw_{c}t},$$

where $h_{tx}$ represents the system function that reflects the hardware differences of the radiating source, characterizing the unintentional modulations. $X\left(w\right)$ is the spectrum of the intentionally modulated signal $x\left(t\right)$, which does not contain hardware-specific information. W denotes the signal bandwidth, $w_{c}$ is the carrier frequency, and ∗ denotes the convolution operation.

Before the radio signal $T_{x}\left(t\right)$ is converted into the baseband signal $R_{x}\left(t\right)$ by the receiver, it is subject to interference from various factors such as channel fading, environmental conditions, and multipath effects. These interferences introduce unintentional modulation and signal distortion beyond the intended modulation. The baseband signal $R_{x}\left(t\right)$, affected by these interferences, can be expressed as:

(2) $$R_{x}\left(t\right)=h_{c}*T_{x}\left(t\right)+n\left(t\right),$$

where $h_{c}$ represents the function that models the effects of the interference, and $n\left(t\right)$ denotes the noise. The operational principle is illustrated in Figure 1. By using appropriate methods, the radio frequency fingerprint can be extracted from the signal $R_{x}\left(t\right)$, enabling individual identification. Suppose the interference during the collection of training data is $h_{C}^{\mathrm{train}}$, and the interference in the test dataset is $h_{C}^{\mathrm{test}}$. When $h_{C}^{\mathrm{train}}\neq h_{C}^{\mathrm{test}}$, the distribution of the training and test data becomes inconsistent, leading to model overfitting on the training set and degraded performance on the test set. We aim for the proposed radio frequency fingerprint identification method to extract hardware-specific radio frequency fingerprints from the signal $R_{x}\left(t\right)$ and to be robust (or resilient) to interference, potentially even eliminating its impact entirely.

## 4. System Description and Preprocessing

### 4.1. System Structure

**Figure 2:**
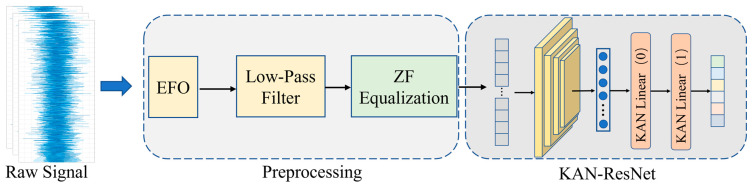
The overall framework of the proposed RFFI method.

Figure 2 illustrates the framework structure of the proposed method. The raw I/Q signals are firstly preprocessed, including the extraction of the L-STF and L-LTF preamble fragments of the steady-state signals, estimation of frequency offset (EFO), and low-pass filter demodulation, in order to realize a reduction in noise in the signal, and to compensate for the channel-induced distortion, so as to improve the recovery accuracy of the signal. Then, ZF equalization of the processed preamble signals is accomplished in preprocessing to resist channel distortion caused by the undesirable channel and to remove ISI, which removes the effect of the time-varying channel on the later network when extracting the feature and classifying the signals.

Preprocessed time-domain raw I/Q signals are input to the KAN-ResNet model. The RFF features are first extracted by the residual block and then classified using the KANLinear layer. By combining the B-spline base function and the SiLU activation function, the KANLinear layer is able to realize the nonlinear mapping of the complex function. This enhances the model’s ability to approximate the complex function and capture the complex features of the RF signal. The generalization ability and recognition accuracy of the KAN-ResNet network are further enhanced by dynamic mesh updating and regularization loss. The data preprocessing module is described in detail as follows.

### 4.2. Preprocessing

**Figure 3:**
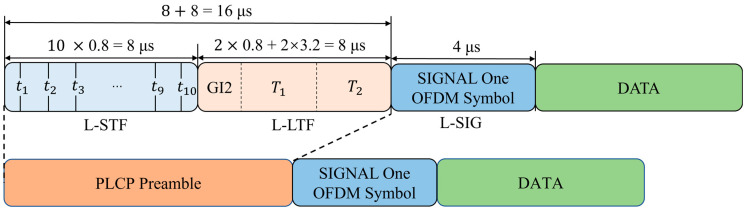
PPDU frame format and PLCP preamble signal.

In selecting the signal, we chose the preamble signal from the Physical Layer Convergence Protocol (PLCP) Protocol Data Unit (PPDU) defined in the IEEE 802.11a standard, as shown in Figure 3.

According to the IEEE 802.11a protocol, this section primarily includes two signal segments: L-STF and L-LTF, as shown in Figure 3. L-STF is the legacy short training field consisting of 10 short symbols (8 us). L-LTF is the legacy long training field consisting of the training symbol guard interval (GI2) and two long symbols (8 us). L-SIG is the legacy signal field (4 us). L-STF is mainly used for coarse CFO estimation, while L-LTF is mainly used for fine CFO estimation. The raw I/Q signal segments identified by the following network are selected as L-STF and L-LTF. The reason for selecting this segment is that it belongs to the steady-state signal, making it easier to capture, and its length and structure remain unchanged regardless of the subcarrier modulation scheme. Additionally, since these two signal segments transmit fixed content, the accuracy of RFF identification is not affected by changes in signal content.

Before performing signal equalization, a coarse frequency offset estimate is first introduced into the L-STF segment to provide an initial frequency correction, frame synchronization, and timing estimation. Subsequently, a refined EFO is applied to the L-LTF segment to correct signal distortion caused by frequency deviation and channel effects. This process enhances communication reliability and demodulation accuracy. Next, a low-pass filter is used to demodulate the L-LTF signal to estimate noise variance and channel response. These two steps help suppress noise enhancement, ensure signal quality, and compensate for distortion caused by the channel, ultimately improving the accuracy of signal recovery.

### 4.3. ZF Equalization

The fundamental principle of channel equalization is to apply an inverse process at the receiver to counteract channel distortion caused by non-ideal transmission. Similar to inverse filtering, it helps mitigate ISI and ensures accurate signal recovery.

Unlike MMSE equalization, which is based on knowledge of the channel and noise, ZF equalization can better reduce ISI caused by multipath effects, and its algorithm is relatively simple with a lower computational complexity. As shown in Equation (2), the transmitted signal $T_{x}\left(t\right)$ is affected by various factors, including the channel, leading to differences between the transmitted and received signals, $R_{x}\left(t\right)$. The core idea of ZF equalization is to find a matrix W such that the equalized signal $\hat{T_{x}}\left(x\right)$ closely approximates the original signal $T_{x}\left(t\right)$, i.e.,

(3) $$\hat{T_{x}}\left(x\right)=W\cdot R_{x}\left(t\right),$$

To reduce channel effects, the matrix W must satisfy $W\cdot h_{c}\approx I$ (where I is the identity matrix). Ideally, the ZF equalizer selects W as the pseudo-inverse of the channel matrix $h_{c}^{-1}$ denoted as:

(4) $$W=h_{c}^{*},$$

where $h_{c}^{*}$ represents the pseudo-inverse of the channel matrix $h_{c}$. By multiplying the received signal $R_{x}\left(t\right)$ with the ZF equalizer W, we obtain:

(5) $$\hat{T_{x}}\left(x\right)=h_{c}^{*}\cdot R_{x}\left(t\right)=h_{c}^{*}\cdot \left(h_{c}*T_{x}\left(t\right)+n\left(t\right)\right),$$

(6) $$\hat{T_{x}}\left(x\right)=T_{x}\left(t\right)+h_{c}^{*}\cdot n\left(t\right),$$

and in ideal conditions (i.e., in the absence of noise), since $h_{c}^{*}\cdot h_{c}=I$, the original signal $T_{x}\left(t\right)$ can be fully recovered, effectively removing ISI. Therefore, ZF equalization can efficiently remove the interference caused by multipath effects, providing clearer data for deep learning models.

**Figure 4:**
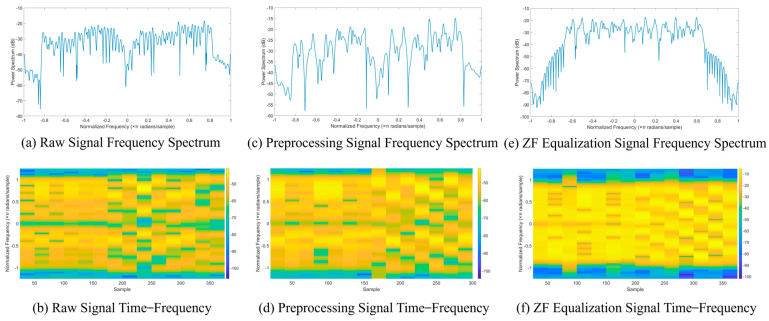
The spectrogram and time-frequency representation of signals before and after processing. The horizontal coordinate of the spectrogram is the normalized frequency in ×π radians/sample; the vertical coordinate is the frequency spectrum in dB. Time-frequency graphs have the horizontal coordinate of the sample; the vertical coordinate is the normalized frequency in ×π radians/sample.

Next, by examining the spectrum and time-frequency diagrams of the Wi-Fi signal before and after equalization in Figure 4, we can intuitively observe the importance of equalization for RFFI. Figure 4a,b show the spectrum and time-frequency diagrams of the unequalized Wi-Fi preamble signal, while Figure 4c,d represent the preprocessed signal. Figure 4e,f illustrate the spectrum and time-frequency diagrams after zero-forcing equalization. After equalization, much of the noise and interference is removed, preserving as many RFF features as possible.

## 5. The Proposed Model of KAN-ResNet

**Figure 5:**
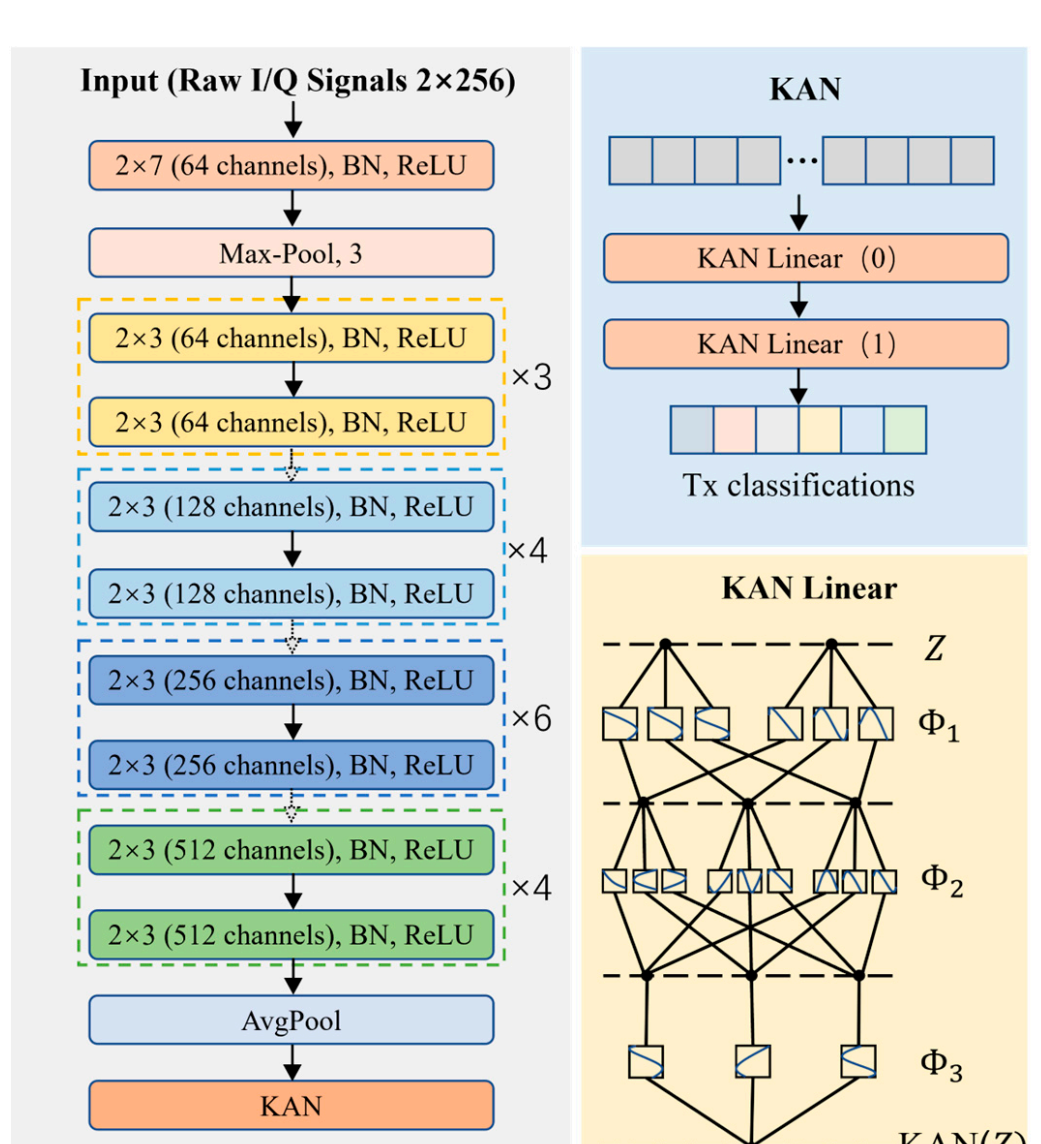
Architecture of the proposed KAN-ResNet, where Z is the activation value and $\Phi_{1},\Phi_{2},\Phi_{3}$ are the activation functions.

Figure 5 illustrates the overall architecture of the proposed KAN-ResNet model, as shown in the KAN-ResNet section of Figure 2. The input signal first passes through residual blocks for feature extraction, followed by prediction and classification using the KAN block. The residual blocks consist of two 2 × 3 convolutional layers, while the KAN block is composed of two KANLinear layers. This network combines the strengths of ResNet [46] and KANs [40]. It not only maintains the residual network powerful feature extraction capability, but also enhances the model’s ability to approximate complex functions and capture intricate features of RF signals by replacing traditional fully connected layers with the KAN model. The goal is to perform complex feature combinations using learnable nonlinear functions rather than relying on simple linear functions, thereby improving the accuracy and generalization capability of the classification tasks.

### 5.1. Feature Extraction Stage

The feature extraction process is primarily composed of multiple BasicBlock residual blocks. These residual blocks are designed with skip connections, significantly reducing the difficulty of training deep networks while effectively avoiding the vanishing gradient problem. Each residual block contains two convolutional layers, followed by batch normalization (BN) and Rectified Linear Unit (ReLU) activation functions. These designs enable the model to extract high-level features from the input radio frequency signal, and ensure good training performance even as network depth increases.

Each BasicBlock residual block contains two convolutional layers, as shown in Figure 5. Each convolutional layer uses a 2 × 3 kernel with a stride of 1 and padding of 1. This configuration allows the convolution operation to extract local features without altering the size of the feature map. The batch normalization layer stabilizes the distribution of the convolutional output, reducing the risk of vanishing or exploding gradients during training. The ReLU activation function introduces nonlinearity, enhancing the expressive power of network. Each residual block employs a skip connection, which directly passes the input to the output of the block and adds it to the output after two convolutional layers. This design learns the residual (that is, changes) between the input and output while retaining the original input information. Skip connections not only reduce the difficulty of training deep networks, but also prevent gradient degradation, enabling the network to effectively train deeper layers.

Between different residual blocks, down-sampling layers are introduced to adjust the feature map size to match the input dimensions of the next convolutional layer. Down-sampling is typically achieved using a 1 × 1 convolution with a stride of 2, which reduces the spatial dimensions of the feature map and adjusts the number of channels. This operation compresses the size of the feature map, increases computational efficiency, and retains important information about the feature.

At the final stage of feature extraction, the model incorporates an adaptive average pooling layer to further compress the feature map to a fixed size (usually 1 × 1). This step not only performs dimensionality reduction in preparation for the KAN module but also preserves global feature information.

### 5.2. KAN as Efficient Classifier

The objective of this study is to introduce KANs into the residual network framework and explore their potential in RFFI. KANs offer advantages in terms of efficiency and interpretability, making them unique compared to traditional MLPs in model design. An MLP composed of N layers can be represented as the interaction between the transformation matrix W and the activation function σ, with the mathematical expression as follows:

(7) $$MLP\left(x\right)=\left(W_{N-1}\;\sigma \;W_{N-2}\;\sigma \;\ldots \;W_{1}\;\sigma \;W_{0}\right)Z,$$

Although an MLP achieves complex function mapping through multiple layers of nonlinear transformations, its inherent opacity hinders model interpretability and presents challenges in understanding the decision-making mechanism. To address the limitations of MLPs in terms of parameter efficiency and interpretability, Liu et al. [40] proposed the KAN, inspired by the Kolmogorov–Arnold representation theorem [47]. Similar to an MLP, an N-layers KAN can be described as a nested structure of multiple KAN layers, with the mathematical expression as follows:

(8) $$KAN\left(Z\right)=\left(\Phi_{N-1}\;\Phi_{N-2}\;\ldots \;\Phi_{1}\;\Phi_{0}\right)Z,$$

where $\Phi_{i}$ represents the i-th layer of the KAN network. Each layer of the KAN consists of a learnable activation function Φ, with $n_{\mathrm{in}}$ dimensional input and $n_{\mathrm{out}}$ dimensional output.

(9) $$\Phi =\left\{\varphi_{q,p}\right\},\;p=1,2,\ldots ,n_{in},\;q=1,2,\ldots ,n_{out},$$

The values of the *n* + 1-th layer can be viewed as a matrix that represents a function of all activation values from the *n*-th layer, as shown below:

(10) $$Z_{n+1}=\left(\begin{array}{ccc} \Phi_{n,1,1}(\cdot ) & \cdots & \Phi_{n,1,k_{n}}(\cdot )\\ \vdots & \ddots & \vdots \\ \Phi_{n,k_{n+1},1}(\cdot ) & \cdots & \Phi_{n,k_{n+1},k_{n}}(\cdot ) \end{array}\right)Z_{n},$$

For a network composed of N layers with k neurons per layer, where $\left(n,i\right)$ represents the i-th neuron in the n-th layer, the activation value is denoted as $x_{n,i}$. Between the n-th and n+1-th layer, there are $n_{k}*n_{k+1}$ activation functions. The activation function connecting the neurons $\left(n,i\right)$ and $\left(n+1,j\right)$ can be defined as:

(11) $$\Phi_{n,i,j},\;n=0,\ldots ,N-1,\;i=1,\ldots ,k_{n},\;j=1,\ldots ,k_{n+1},$$

The pre-activation value of the activation function $\Phi_{n,j,i}$ is $x_{n,i}$, and the post-activation value ${\tilde{x}}_{n,j,i}$ is defined as ${\tilde{x}}_{n,j,i}=\Phi_{n,j,i}\left(x_{n,i}\right)$. The activation value of neuron $\left(n+1,j\right)$ can be simplified as the sum of all post-activation values:

(12) $$x_{n+1,j}=\sum_{i=1}^{k_{n}}{\tilde{x}}_{n,j,i}=\sum_{i=1}^{k_{n}}\Phi_{n,j,i}\left(x_{n,i}\right),\;j=1,\ldots ,k_{n+1},$$

thus, the values of the n+1-th layer can be viewed as a matrix that represents the activation values of the n-th layer, as shown in Equation (10).

While Equation (12) appears simple, optimizing this structure is challenging. The key lies in setting up the residual activation function and updating the spline grid. The activation function $\Phi_{x}$ is composed of the basis function $b\left(x\right)$ and the spline function $spline\left(x\right)$:

(13) $$\Phi \left(x\right)=w_{b}b\left(x\right)+w_{s}spline\left(x\right),$$

and the basis function is typically defined as:

(14) $$b\left(x\right)=\mathrm{silu}\left(x\right)=\frac{x}{1+e^{-x}},$$

where $\mathrm{silu}\left(x\right)$ is the Sigmoid Linear Unit activation function. The spline function is parameterized as a linear combination of B-splines:

(15) $$spline\left(x\right)=\sum_{i}c_{i}B_{i}\left(x\right),$$

where $c_{i}$ represents the corresponding coefficients, which are trainable, and $B_{i}\left(x\right)$ is the i-th B-spline basis function. In principle, $w_{b}$ and $w_{s}$ are redundant since they can be absorbed into $b\left(x\right)$ and $spline\left(x\right)$, but they are retained (and by default, trainable) to provide better control over the overall amplitude of the activation function. During initialization, $w_{s}$ is set to 1 and $spline\left(x\right)$ is set to 0. The spline grid needs to be dynamically updated based on input activations to account for the fact that splines are defined over a finite region, while activation values may exceed this fixed region during training.

### 5.3. KANLinear Module

Traditional fully connected layers play a crucial role in neural networks, but their linear structure and fixed activation functions have limitations when processing complex data. To enhance the computational efficiency and expressive power of the network, this study designs the KANLinear module based on the principles of KANs, as shown in Figure 5, to replace the fully connected layers in traditional residual networks. By combining B-spline basis functions with traditional activation functions, this module achieves complex nonlinear mappings. Compared to the traditional Fully Connected (FC) layer, which relies on a single fixed activation function (e.g., ReLU or Sigmoid), the KANLinear module improves modeling capability in several ways. First, it combines learnable B-spline basis functions with simpler activation functions (e.g., SiLU), allowing each basis function to adapt to the training data, thus capturing the subtle nonlinear properties of RFF signals. Second, since these B-splines are explicitly represented, KANLinear offers greater interpretability than the “black box” weight matrices in the FC layer. Finally, the module’s dynamic mesh update mechanism adjusts the spline nodes in real time to track variations in input distributions—an essential advantage in time-varying channels.

#### 5.3.1. B-Spline Activation Function

The core idea of the KANLinear layer is to replace the traditional linear weight matrix with B-spline basis functions, as shown in Equation (15). This method allows the neural network to apply learnable nonlinear transformations to each input feature, significantly enhancing the flexibility of model. In traditional implementations, all intermediate variables must be expanded to apply different activation functions, resulting in high memory consumption. The KANLinear module reduces memory requirements by introducing B-spline basis functions and linearly combining them with the base activation function, as described in Equation (13).

B-splines are basis functions used for function approximation and interpolation, known for their locality, smoothness, and numerical stability. In the KANLinear model, B-spline basis functions are used for the nonlinear transformation of the input tensor, thereby improving the expressive power of model. Specifically, B-spline basis functions perform interpolation and approximation on the input data over a given grid, enabling complex nonlinear transformations.

Definition: Given a B-spline basis function $B_{i,k}\left(x\right)$ of order k,the recursive process is as follows. The initialization is defined as:

(16) $$B_{i,0}(x)=\left\{\begin{array}{cc} 1, & \;\;if\;t_{i}\leq x<t_{i+1}\\ 0, & \;\;otherwise \end{array}\right.,$$

where $t_{i}$ is the i-th point in the grid. The recursive relation is given by:

(17) $$B_{i,k}\left(x\right)=\frac{x-t_{i}}{t_{i+k}-t_{i}}B_{i,k-1}\left(x\right)+\frac{t_{i+k+1}-x}{t_{i+k+1}-t_{i+1}}B_{i+1,k-1}\left(x\right),$$

which indicates that a B-spline basis function of order k can be expressed as a linear combination of two B-spline basis functions of order k−1.

#### 5.3.2. Calculation of Spline Basis Function Weights and Curve Interpolation

To achieve nonlinear data combinations, the model computes the weights of the B-spline basis functions for interpolation. This process involves solving a system of linear equations to find a set of coefficients that allow the B-spline basis functions to approximate the output at the given input points.

Mathematical description: Given input data X and output data Y, we need to find a matrix W such that:

(18) $$Y=WB\left(X\right),$$

where $B\left(X\right)$ is the B-spline basis function matrix. By solving this using the least squares method, the weight matrix W can be defined as:

(19) $$W={\left(B^{T}B\right)}^{-1}B^{T}Y$$

#### 5.3.3. Grid Generation and Updating

The grid determines the position of the B-spline basis functions, and in the KANLinear module, the generation and updating of the grid directly affect the shape and coverage of the spline basis functions. The initial grid is defined as:

(20) $$h=\frac{\left(x_{\max}-x_{\min}\right)}{g_{s}},$$

(21) $$grid=\{t_{i}|t_{i}=h\cdot i+x_{\min},i=-n,-n+1,\ldots ,g_{s}+n\},$$

where h represents the grid step size, $x_{\min}$ and $x_{\max}$ are the lower and upper bounds of the grid, respectively, $g_{s}$ denotes the grid size, $t_{i}$ is the i-th point in the grid, and n represents the spline order.

Next, the positions of the grid points are dynamically adjusted based on the input data. By combining uniform and adaptive grids, the updated grid is obtained as:

(22) $$t_{i}^{\mathrm{updated}}=\epsilon \cdot t_{i}^{\mathrm{uniform}}+\left(1-\epsilon \right)\cdot t_{i}^{\mathrm{adaptive}},$$

where $t_{i}^{\mathrm{uniform}}$ is the *i*-th grid point generated by a uniform distribution, $t_{i}^{\mathrm{adaptive}}$ is the i-th grid point generated adaptively, and the parameter ϵ controls the weighting between the uniform and adaptive grids. The adjusted grid allows for a more precise capture of local features in the input data, thereby improving the fitting ability of the model.

#### 5.3.4. Regularization Loss

In the original implementation, L1 regularization required nonlinear operations on tensors, which is not suitable for the redesigned computational structure. In this study, we replace the traditional regularization method with L1 regularization on the weights, divided into two parts.

Activation Regularization: This calculates the sum of the average absolute values of all spline weights:

(23) $${Loss}_{ac}=\sum \left|W_{s}\right|,$$

Entropy Regularization: This computes the probability $p_{i}$ of each weight relative to the total sum, followed by the negative entropy:

(24) $$p_{i}=\frac{\left|w_{i}\right|}{\sum \left|w_{i}\right|},$$

(25) $$Loss_{en}=-\sum p_{i}\log p_{i},$$

and the final regularization loss is the weighted sum of activation regularization and entropy regularization:

(26) $$Loss_{reg}=\lambda_{1}Loss_{ac}+\lambda_{2}Loss_{en},$$

where $\lambda_{1}$ and $\lambda_{2}$ are regularization hyperparameters. To optimize the network’s performance on the Wi-Fi signal dataset, KANLinear utilizes the Kaiming initialization method to ensure the stability of the weight distribution.

#### 5.3.5. Computational Complexity Analysis

The KAN-ResNet network proposed in this study has a primary computational complexity that can be approximated as O(H×W) when the input size is H×W. Under the dataset and hyperparameter settings used in our experiments, the total number of trainable parameters in the model is about 25,999,040, and the actual inference process requires about 3.3 to 3.5 GFLOPs of computational power, depending on the specific implementation details and hardware configurations. Compared to the traditional ResNet architecture, the introduction of B-spline basis functions in KAN-ResNet introduces some computational overhead. However, by replacing some fully connected layers and simplifying the network accordingly, we effectively maintain or even improve the detection accuracy without significantly increasing the overall parameter size.

### 5.4. Model Configuration and Overall Architecture Description

#### 5.4.1. Role of the KAN Module in the Overall Architecture

In our designed network, the traditional fully connected layers are replaced with KAN modules. Specifically, in the final stage of ResNet, we apply the following configuration:

- The KAN module is instantiated using KAN ([512 × block.expansion, 64, num_classes]);
- Input Dimension: After processing through convolution and residual blocks, the feature vector is obtained using adaptive average pooling and flattening. Its size is 512 × block.expansion (in this case, block.expansion = 1, resulting in a 512-dimensional vector);
- Hidden Layer: The first KANLinear layer maps the 512-dimensional input to a 64-dimensional space;
- Output Layer: The second KANLinear layer maps the 64-dimensional features to the output space, corresponding to the number of classes (e.g., the number of transmitters).

#### 5.4.2. Configuration and Implementation Details of the KANLinear Layer

In each KANLinear layer, we apply two transformations to the input data and then combine the results.

1. Basic Linear Transformation: The parameter base_weight is used with a base activation function (default: SiLU) to perform standard linear mapping, expressed as:
  (27) $$base_{output}=F.linear\left(SiLU\left(x\right),base_{weight}\right),$$
  the scaling factor for this part is scale_base (default: 1.0), and Kaiming uniform initialization is applied;

2. In the B-spline(x) computation, the method first utilizes a pre-generated grid, registered as a buffer. This grid is defined by the parameters grid_size (default: 5), which determines the number of intervals in the B-spline grid; spline_order (default: 3), which represents the order of the piecewise polynomial; and grid_range (default: [−1, 1]), which specifies the range of grid values. Using these parameters, the method calculates the grid step size and computes the corresponding B-spline basis functions for each input feature. During the iterative process, it applies weighted summation based on the predefined formula, ultimately forming a basis function matrix of shape (batch_size, in_features, grid_size + spline_order).

In B-spline weights and curve fitting, the parameter spline_weight (shape: [out_features, in_features, grid_size + spline_order]) performs a linear combination of the B-spline basis function outputs, producing spline_output. The curve2coeff method applies least squares optimization to determine the coefficients between the B-spline basis functions and the target function. This process refines the randomly initialized noise (scaling factor: scale_noise, default: 0.1) into meaningful piecewise polynomial weights. If standalone piecewise polynomial scaling is enabled (enable_standalone_scale_spline = True), the method further applies spline_scaler (initialized using Kaiming uniform initialization, scaling factor: scale_spline, default: 1.0) to scale spline_weight.

The final output of the KANLinear layer is obtained by adding the outputs of the basic linear layer and the B-spline nonlinear transformation:

(28) output=base_output+spline_output

Parameter summary:

- grid_size: Determines the number of intervals in the B-spline grid (default: 5);
- spline_order: Defines the polynomial order (default: 3), affecting the smoothness and fitting ability of the nonlinear transformation;
- scale_noise: Controls the scaling of random noise (default: 0.1) for initializing spline_weight;
- scale_base and scale_spline: Control the scaling of the basic linear transformation and B-spline component, respectively (both default to 1.0);
- grid_eps: Adjusts the weight between uniform grid and adaptive grid during updates (default: 0.02);
- grid_range: Specifies the range of grid values (default: [−1, 1]);
- base_activation: The base activation function, with SiLU as the default choice;
- num_classes: It represents the number of classes in the model’s final output, which is set based on the number of transmitters;
- base_weight: It is the learnable weight matrix for the basic linear transformation, initialized using Kaiming uniform initialization;
- batch_size: It represents the number of data samples used during each training session, which is set to 64 in this paper;
- in_features: It specifies the dimension of the input features, i.e., the length of the vector entering a layer, which is determined by the output of the preceding network;
- out_features: It specifies the dimension of the output features for that layer, with the final result being equal to num_classes;
- enable_standalone_scale_spline: It controls whether to enable an independent piecewise polynomial scaling factor, initialized using Kaiming uniform initialization.

#### 5.4.3. Overall Architecture, Processing Steps, and Data Flow Description

**Figure 6:**
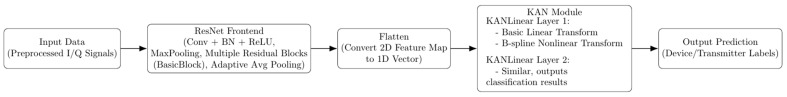
KAN-ResNet model principle flowchart.

The principle flowchart of the KAN-ResNet model is shown in Figure 6, and the data flow throughout the system is as follows.

1. Data Input and Preprocessing: The system collects and preprocesses Wi-Fi signals, including extracting the IEEE 802.11 preamble, frequency offset correction, and low-pass filtering. Finally, the processed data are formatted into a tensor of shape (batch_size, 1, 256, 2);
2. ResNet Front-End Feature Extraction: The data first pass through a 2 × 7 convolutional layer, followed by batch normalization and ReLU activation. A max pooling layer then reduces its size. Next, the data flow through multiple residual blocks (BasicBlock), where each block contains two 2 × 3 convolutional layers and maintains information flow through residual connections. Finally, adaptive average pooling converts the high-dimensional feature maps into a fixed-size representation, which is then flattened into a one-dimensional vector;
3. KAN Module Classification: The extracted 1D feature vector is fed into the KAN module, which consists of multiple KANLinear layers. Each layer performs a basic linear transformation by applying a standard linear mapping to the input and introducing nonlinearity through the SiLU activation function. It then computes the B-spline basis functions based on a predefined grid that can be adaptively updated. Using B-spline weights, it performs a linear combination of the basis function outputs. Finally, the results from the linear transformation and B-spline computation are summed to produce the final output. The KAN module generates the classification result, corresponding to the predicted device or transmitter category;
4. Training and Evaluation: The model is trained using the cross-entropy loss function for supervised learning and optimized with the AdamW optimizer. During training, the update_grid method periodically updates the B-spline grid in the KAN module, allowing it to better adapt to the input data distribution. After training, the model is evaluated on different test sets, including same-day and cross-day data.

## 6. Experiments

After the data preprocessing and equalization described in Section 4, the KAN-ResNet is used for training and classifying the Wi-Fi preamble signal. The overall experimental process includes data preparation, model training, and model evaluation. First, the wireless signal dataset is loaded and prepared, and then split into a training set (80%), validation set (10%), and test set (10%). During the model training phase, the Adam optimizer is employed for parameter updates, and a learning rate scheduler dynamically adjusts the learning rate. The model optimizes the classification task using the CrossEntropyLoss function and introduces an early stopping mechanism, which halts training when the validation loss no longer decreases. Finally, the model is evaluated on the test set to measure its classification accuracy. To verify the generalization ability of model on time-series data, accuracy comparisons are also conducted on test sets from different time periods (e.g., different days).

### 6.1. Datasets and Experiment Setup

#### 6.1.1. Overview of the Datasets

We use the WiSig dataset [23] to verify our proposed RFFI scheme and test it compared to existing methods. A subset of ManySig (2.2 GB) was used, consisting of raw and equalized data from 12 receivers at six transmitters, with four different captures over a one-month period, each one week apart. The data were acquired on March 1st, March 8th, March 15th and March 21st. Each data packet contains 256 I/Q samples collected on a wireless channel with a center frequency of 2462 MHz and a bandwidth of 20 MHz used by IEEE802.11a/g modules. The selection of this dataset is based on its inclusion of multiple devices and multiple collection days, which effectively reflects the impact of environmental variations on RFFI in real-world scenarios.

The other dataset is ManySig-ZF (https://huggingface.co/datasets/bobo-Josef/ManySig_ZF/tree/main (accessed on 13 February 2025)), which uses ZF equalization. The transmitter and receiver types, as well as the data collection dates, are identical to the ManySig dataset. This dataset is 1.46 GB in size. In this study, we replaced the MMSE equalization method for the Wi-Fi signal preamble with the more effective ZF equalization. Before equalization, the raw signals were preprocessed using Matlab, including extracting the preamble segment, estimating frequency offset, and calculating noise variance. The preprocessed preamble segments were then equalized, and the results before and after equalization are shown in Figure 4. The processed complex signal in the time domain separates the real part from the imaginary part. It is packaged in Python 3.9 as a ManySig-ZF dataset containing up to 1000 data packets per transmitter.

#### 6.1.2. Experimental Scenario Construction

In the experiment, we used data captured on March 1st, March 8th, and March 15th from the WiSig dataset, which were split into training (80%), validation (10%), and testing (10%) sets. Three training scenarios were considered: using only March 1st data (denoted as D1), using both March 1st and March 8th data (denoted as D2), and using March 1st, March 8th, and March 15th data (denoted as D3).

The testing was divided into two categories:

- Same-Day Testing: For same-day testing, both training and testing data were taken from the same capture day. Specifically, after training on March 1st data, testing was performed on March 1st data; after training on March 1st and March 8th data, testing was conducted on data from these two days; and after training on data from March 1st, March 8th, and March 15th, testing was carried out on data from these three days;
- Different-Days Testing: For different-days testing, the training data corresponded to one of the D1, D2, or D3 scenarios, while the testing data were uniformly taken from the March 21st capture.

In total, six testing scenarios were established (three for same-day testing and three for different-days testing), as detailed in Table 1.

This design is intended to simulate real-world Wi-Fi signal variations, where changes over time caused by environmental factors (e.g., temperature, humidity, and multipath effects), device aging, and other practical factors lead to variations in signal characteristics. By comparing the results of same-day and different-days testing, we can directly assess the impact of data collection time differences on RFF identification accuracy and verify the robustness and practicality of our proposed method in realistic scenarios.

#### 6.1.3. Experimental Platform and Training Details

The experiments were conducted in an Ubuntu environment, with deep learning models built using the PyTorch framework. Software used included: Visual Studio Code (version 1.85), PyCharm 2023.3.2, MATLAB R2022a. The optimizer used was Adam. Detailed hardware specifications included an Nvidia Tesla V100 with 32 GB of VRAM. The learning rate was initially set to 0.001 during training, with a batch size of 64. If the validation accuracy did not increase within the last five epochs, training was stopped to prevent overfitting. To examine the impact of data collected on different days on RFFI accuracy, data from the first three days were used for training and validation (source domain), and data from the final day were used for testing (target domain). Additionally, the potential of this method in practical applications was demonstrated by recording the average accuracy. Finally, the superiority of our approach was highlighted through comparisons with several state-of-the-art neural networks.

### 6.2. Experimental Results and Analysis

A total of three experiments were conducted. In Experiment 1, Wi-Fi signals that underwent only preprocessing were trained and tested using the WiSigCNN [23], KAN-ResNet, and ResNet_FC (fully connected layer) networks. This experiment aims to evaluate whether the residual network designed in this study can achieve higher accuracy compared to WiSigCNN. In Experiment 2, MMSE-equalized Wi-Fi signals were used for identification to explore the improvement in performance of KAN-ResNet on equalized signals. In Experiment 3, the use of neural networks to train and test ZF equalization Wi-Fi signals was evaluated to investigate whether ZF equalization can better reduce the effects of time-varying channels on Wi-Fi signals during transmission and propagation compared to MMSE equalization. Various recognition algorithms were compared to study the advantages of the KAN-ResNet model.

#### 6.2.1. Experimental Results of Unequalized Wi-Fi Signals

**Figure 7:**
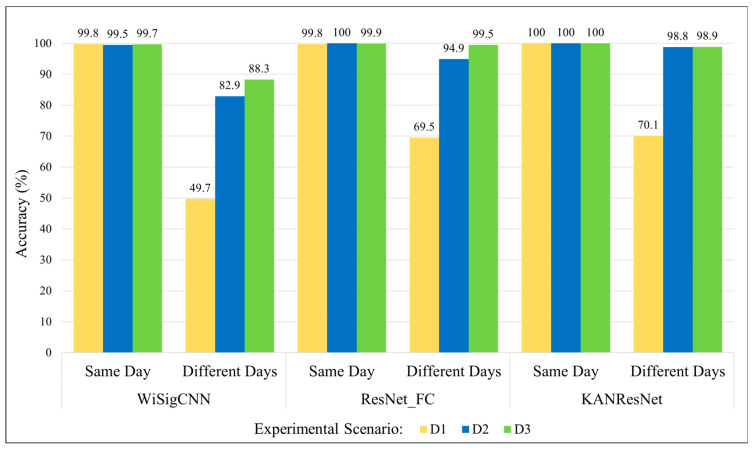
Experimental classification accuracy of unequalized data in different experimental scenarios.

The data for unequalized Wi-Fi signals used in this experiment come from the ManySig subset of the WiSig dataset. The results of the experiment are shown in Figure 7. The results demonstrate that as the time interval between data collection increases and the number of training days decreases, the accuracy of the model on test data from different days significantly declines. This indicates that Wi-Fi signals are heavily affected by environmental factors and multipath effects during transmission and propagation. Although replacing the network model and increasing the number of training days can help the model learn more RFF features and increase RFFI accuracy, this approach requires frequent collection of Wi-Fi data from the transmitter and retraining the model. This process increases costs in practical applications and places storage demands on the device, significantly limiting the practical value of this technology.

Moreover, Figure 7 shows that the KAN-ResNet designed in this study achieves higher accuracy in different-days tests compared to the WiSigCNN and ResNet_FC networks. For example, in the case of the training data of a single day, KAN-ResNet improves the different-days testing accuracy from 49.7% (WiSigCNN) to 70.1%, which preliminarily validates the effectiveness of the proposed network in handling channel variations.

#### 6.2.2. Experimental Results on MMSE-Equalized Signals

When conducting experiments on MMSE-equalized signals, the proportions for the training, validation, and test sets are the same as for the unequalized signals. The experiments are again divided into same-day and different-days scenarios, with six test cases in total. WiSigCNN, ResNet_FC, and KAN-ResNet models are used to conduct five independent experiments on the equalized data. Based on the standard deviation from these three experiments and the t-distribution critical value at the 95% confidence level, the confidence interval is calculated, and the results are shown in Table 2.

By comparing Table 2 with the results for unequalized data in Figure 7, it can be observed that after equalization, the testing accuracy of neural networks on signals collected from different days improved by at least 20.0% in the training scenario D1. Specifically, the testing accuracy of the WiSigCNN increased from 49.7% before equalization to 93.7%, nearly doubling. This demonstrates that the signals of Wi-Fi transmitters should be equalized before their individual identification. The effect of time-varying channels on RFFs can be effectively reduced, thus improving the accuracy of neural networks in RFF detection and increasing the practical value of this technique.

**Figure 8:**
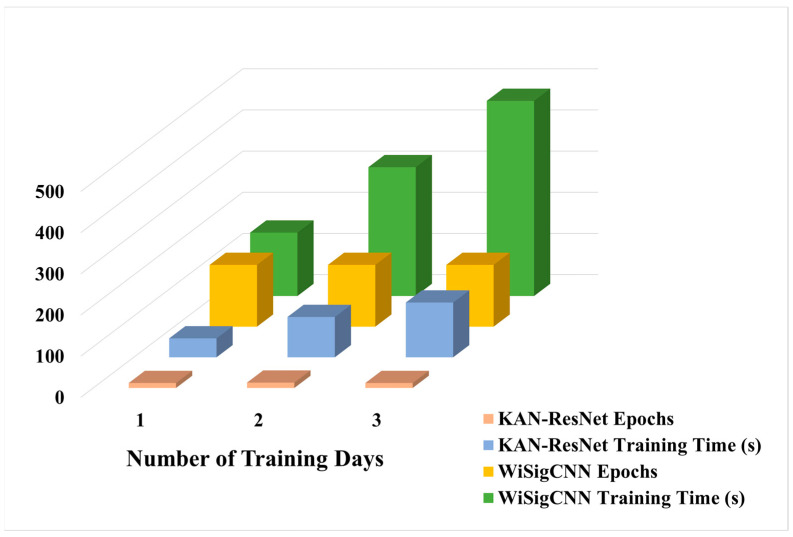
Comparison of training epochs and times between KAN-ResNet and CNN.

With respect to the training scenario D1, WiSigCNN, ResNet_FC, and KAN-ResNet models were tested on different days of data with the experimental results of 93.7%, 94.6%, and 97.6%, respectively. Among them, our proposed KAN-ResNet model has the best accuracy. Notably, by replacing fully connected layers with the KAN module, the number of network parameters was reduced from 26,002,118 to 25,999,040, a reduction of 3078 parameters. Additionally, the KAN-ResNet model required 12, 13, and 12 epochs for the D1, D2, and D3 training scenarios, with training times of 46 s, 98 s, and 133 s, respectively. In contrast, the WiSigCNN trained for 150 epochs under the same conditions, with training times of 154 s, 313 s, and 474 s, as shown in Table 3 and Figure 8. Comparatively, the KAN-ResNet not only required shorter training times but also achieved higher testing accuracy. Although the test time of the KAN-ResNet model is slightly longer than that of WiSigCNN, the difference is less than one second, making it negligible in practical applications.

Experimental results show that KAN-ResNet requires certain hardware performance capabilities. However, its strong feature representation improves accuracy in time-varying channels and diverse environments. In practical applications, it enhances robustness to time-varying channels and enables precise transmitter identification. When accuracy and security demands are high, the additional computational overhead is both acceptable and necessary.

#### 6.2.3. Experimental Results on ZF-Equalized Data

After data preprocessing, the Wi-Fi signal preamble segments were equalized using the method described in Section 3 and repackaged into a new dataset, ManySig-ZF.pkl, with a size of 1.46 GB. The dataset includes the same transmitters, receivers, number of signals, and collection days as the ManySig dataset. In the experiment, the proportions of the training, validation, and test sets, as well as the test setup, are the same as for the unequalized data. To demonstrate the generalizability of the proposed method, the equalized technique is tested on the WiSigCNN, ResNet_FC, and KAN-ResNet models. Each model undergoes three independent experiments on the equalized data. The confidence interval is calculated based on the standard deviation of these experiments and the t-distribution critical value at the 95% confidence level. The results are shown in Table 4.

Compared to the MMSE-equalized test results in Table 2, the WiSigCNN, ResNet_FC, and KAN-ResNet models all improve the test results for the transmitters’ Wi-Fi signals collected on March 21^st^, while our proposed KAN-ResNet model achieves 99.4% accuracy, when tested on Wi-Fi signals from different days after training on only one day of data (in this scenario, the variability between signals from the same transmitter is highest, making identification more challenging). This indicates that ZF equalization is more effective at mitigating the impact of multipath effects on RFF identification, while also offering a simpler algorithm with higher practical value.

According to the results in Table 4, the ResNet_FC outperformed the WiSigCNN in terms of recognition accuracy on Wi-Fi signals from different days, indicating that the ResNet_FC is more adept at extracting RFF features. Furthermore, the proposed KAN-ResNet achieved an accuracy of 99.4% when trained in the D1 training scenario and tested on March 21st data, significantly outperforming the 95.5% of WiSigCNN. Even when trained in the D3 training scenario and tested on March 21st, the accuracy reached 99.9%. Replacing the traditional ReLU activation function with a B-spline function, as shown in Equation (15) in Section 4, transforms the activation function from a complex function to a combination of several learnable simple functions. This not only reduces the number of network parameters but also enhances the ability of model to predict on test samples, thus improving the accuracy of network in identifying Wi-Fi signal RFFs. Additionally, optimizing the regularization function through Equation (26) further enhances the robustness of network.

A statistical analysis is conducted on the independent experimental results under different scenarios in Table 2 and Table 4 (e.g., D1, D2, D3, Same Day, and Different Days). First, the 95% confidence interval is calculated using the mean and standard deviation from five repeated experiments. The results show that KAN-ResNet consistently achieves higher accuracy intervals with less overlap compared to other methods. Next, the paired *t*-test results (*p* < 0.05 or *p* < 0.01) confirm that the accuracy differences between KAN-ResNet, WiSigCNN, or ResNet_FC are statistically significant, indicating that the accuracy improvement observed in different-days testing is not due to random fluctuations. Overall, these findings demonstrate that KAN-ResNet exhibits greater stability and generalization across different equalization conditions (MMSE or ZF), significantly enhancing its practical value for RFFI in wireless environments with large time varying.

By analyzing Table 5, Table 6 and Table 7, we observe that for Wi-Fi signals affected by time-varying channels, ZF equalization significantly improves the model performance. Specifically, the KAN-ResNet network achieves higher precision, recall, and F1-score not only compared to the traditional CNN model, but also compared to the residual network with fully connected layers. Considering the overall experimental results, it is evident that the combination of ZF equalization and KAN-ResNet provides an effective solution to mitigate the impact of time-varying channels in RFFI.

### 6.3. Comparison of ZF and MMSE Equalization Performance in Noisy Environments

To evaluate the performance of ZF and MMSE equalization under real-world noise and multipath conditions, this study designs a noise-enhanced experiment. First, Gaussian white noise is added to the original Wi-Fi signals at different signal-to-noise ratio (SNR) levels (5 dB, 10 dB, 15 dB, and 20 dB). The signals then undergo equalization using ZF and MMSE equalization, respectively. Finally, the equalized signals are input into the KAN-ResNet model for RFFI. This experiment considers three training scenarios (D1, D2, and D3) to simulate different amounts of training data and different time spans. The objective is to assess the overall performance of equalization methods under time-varying channels and different noise conditions.

**Figure 9:**
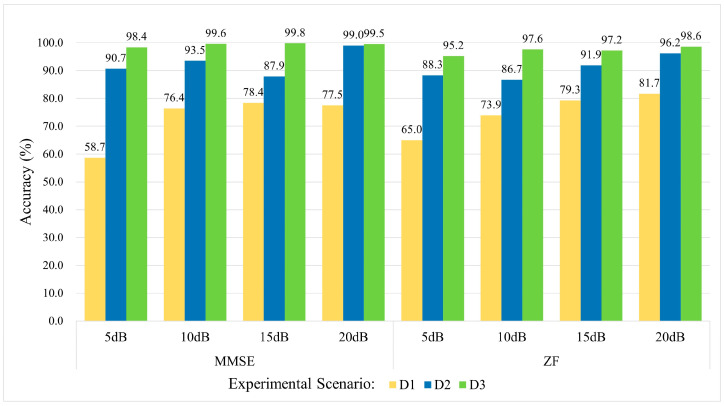
Impact of ZF vs. MMSE equalization on RFFI accuracy across various SNR levels.

As shown in Figure 9, the experimental results indicate that as SNR increases, both ZF and MMSE equalization achieve significant improvements in recognition accuracy. At 20 dB, the performance gap between the two methods is minimal. However, under low SNR conditions (5 dB) with severe channel fading, ZF equalization suffers from noise amplification, leading to relatively lower accuracy. In contrast, MMSE equalization demonstrates greater robustness by balancing noise suppression and ISI removal. The impact of ZF equalization is more pronounced in the D1 scenario, where training data are limited and the time span is longer. In the D2 and D3 scenarios, performance becomes more similar to MMSE equalization. These results suggest that ZF equalization performs well under moderate to high SNR and when the training dataset is sufficient. However, in low SNR conditions or when channel estimation is inaccurate, ZF equalization is more susceptible to noise amplification.

Overall, ZF equalization remains an attractive option due to its simplicity and effectiveness in eliminating ISI. It is well-suited for most scenarios with moderate to high SNR, while MMSE equalization is more appropriate for environments with severe fading or extremely high noise levels. For RFFI in time-varying channels, achieving a balance between noise resistance and computational efficiency requires an adaptive approach. ZF equalization performs well in moderate to high SNR conditions, but in extremely harsh environments, switching to MMSE equalization or adopting a hybrid adaptive strategy helps mitigate the noise amplification effect of ZF equalization. This comparative study provides a more comprehensive evaluation of ZF equalization limitations. It also offers valuable insights for optimizing equalization strategies and model performance in dynamic and complex RFFI environments.

### 6.4. B-Spline Grid Sensitivity Analysis and Experimental Evaluation

The proposed model constructs a B-spline grid that covers the input data range and extends by spline_order step sizes on both sides. This design enables efficient basis function computation and parameter updates. First, the step size is determined based on the predefined grid range and size. The generated grid is then registered as a model buffer to compute the B-spline basis functions corresponding to each input feature. Next, the least squares method is used to solve for the coefficients of the piecewise polynomial, optimizing memory usage for extended activation function computation. To improve adaptability to input data distribution, the model employs a weighted combination of adaptive and uniform grids based on data sorting. The parameter grid_eps controls the weighting between these two components, ensuring grid updates and corresponding spline weight adjustments while maintaining the support conditions. This approach improves the model’s expressive capability and generalization performance.

To investigate the impact of the B-spline grid mechanism on model convergence speed and classification accuracy, we design a comparative experiment by adjusting spline_order and grid_eps. This experiment evaluates how changes in grid parameters affect the training process and generalization performance of KAN-ResNet.

**Figure 10:**
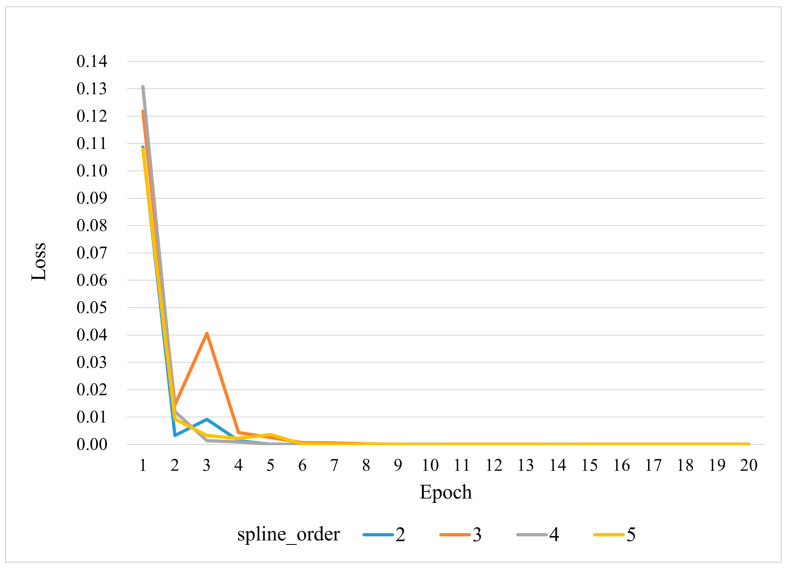
Training loss curves for various spline_order values (2, 3, 4, 5).

In the experiment analyzing the impact of spline_order on convergence speed, grid_eps is fixed at 0.02 (the default value), while spline_order is set to 2, 3, 4, and 5, respectively. The loss curves during training are compared, as shown in Figure 10. The results indicate that while all models experience a rapid loss reduction within the first 1–3 epochs, models with spline_order=3 or 4 converge faster to a stable near-zero loss. This suggests that these settings retain sufficient nonlinear representation capability while effectively avoiding excessive oscillations. When spline_order=2, the initial convergence speed is relatively high, but its fine approximation ability is somewhat limited in later stages. In contrast, spline_order=5 exhibits greater fluctuations in the early phase, which may require stronger regularization or longer training to prevent overfitting. These findings indicate that under the default data size and hyperparameter settings, spline_order=3 or 4 achieves an optimal balance between convergence efficiency and model stability.

In the experiment examining the impact of grid_eps on generalization accuracy, spline_order is fixed at 3 (the default value), while grid_eps is set to 0.01, 0.02, 0.05, and 0.1. The classification accuracy of the model is evaluated across the D1, D2, and D3 scenarios (as shown in Table 8). The results indicate that grid_eps=0.02 achieves the best accuracy across all scenarios (D1: 99.4%, D2: 99.8%, D3: 99.9%), outperforming other values. When grid_eps is too small (0.01) or too large (0.1), recognition accuracy declines in certain scenarios. This could be due to the following reasons:

- When grid_eps is too small (0.01), the adaptive grid fails to sufficiently cover the input data distribution, limiting the model’s nonlinear approximation ability when encountering new or distribution-shifted samples in the test set;
- When grid_eps is too large (0.1), the grid updates too frequently or expands excessively, leading to less precise feature fitting in some scenarios (e.g., D1 or D2) or instability that affects accuracy.

The findings suggest that B-spline grid parameters significantly influence the training and testing performance of KAN-ResNet. Selecting an appropriate spline_order (e.g., 3 or 4) ensures a balance between expressive power and training convergence speed. Similarly, a moderate grid_eps (e.g., 0.02) allows adaptive coverage of activation distributions while maintaining stable approximation, leading to higher recognition accuracy across different training data sizes and time-varying environments. If spline_order or grid_eps deviates from the optimal range, it may cause convergence fluctuations or degradation in generalization performance.

### 6.5. KAN-ResNet: Limitations and Future Work

In extreme environments, KAN-ResNet maintains good robustness under general time-varying channels but struggles in ultra-low SNR, severe multipath fading, or sudden interference conditions. Its B-spline grid update mechanism relies on stable training samples, making it difficult to adapt to rapid channel variations, which may reduce recognition accuracy. Although the overall parameter count remains reasonable, B-spline computations introduce extra computational and storage overhead due to dynamic grid updates. This challenge becomes significant for resource-constrained IoT devices or real-time applications.

To address these limitations, future work will explore adaptive or online learning mechanisms for real-time B-spline grid updates. Additionally, integrating other equalization strategies, such as MMSE or hybrid equalization, may further enhance robustness. Model pruning and quantization techniques will also be investigated to reduce computational complexity and resource usage, improving generalization and practical deployment in large-scale and extreme environments.

### 6.6. Summary of Experiments

Figure 7 clearly shows that as the interval between training and testing data increases, the accuracy of Wi-Fi RFF identification drops significantly. According to studies by Rehman et al. [36], Gu et al. [37], and He et al. [38], environmental factors and multipath effects interfere with the generation and transmission of radio frequency signals, thereby affecting the neural network’s ability to extract and identify RFF features. Comparing the experimental results in Table 2 and Figure 7, we observe that under the same network and experimental conditions, the testing accuracy of equalized Wi-Fi preamble signals on different days reached a minimum of 93.7%, which is 44.0% higher than that of the unequalized data. Moreover, when we restrict the training data to a single day, the KAN-ResNet model’s testing accuracy improves dramatically from 70.1% to 97.6% (as shown in Table 2) without any additional training time. This demonstrates that equalization is an effective method to mitigate the influence of environmental factors and multipath effects. In particular, it reduces the interference of signal generation (e.g., temperature, pressure) and propagation (e.g., multipath effects) with the RFF.

Although KAN-ResNet reaches 97.6% accuracy on multi-day test sets using MMSE equalization (as shown in Table 2), further improvements are observed with ZF equalization. Specifically, Table 4 shows that switching to ZF equalization enhances Wi-Fi signal accuracy from 97.6% to 99.4%, while the WiSigCNN framework improves from 93.7% to 95.5%. By observing Figure 9, ZF equalization is simpler to implement and requires less computation than MMSE equalization. In medium- to high-SNR scenarios, it effectively eliminates inter-symbol interference and achieves performance similar to MMSE. When training data are sufficient and channel estimation errors are small, its noise amplification issue is not significant. Therefore, ZF equalization remains a strong choice for most applications with moderate or high SNR. These results indicate that ZF equalization is more effective than MMSE in removing ISI caused by multipath effects.

**Figure 11:**
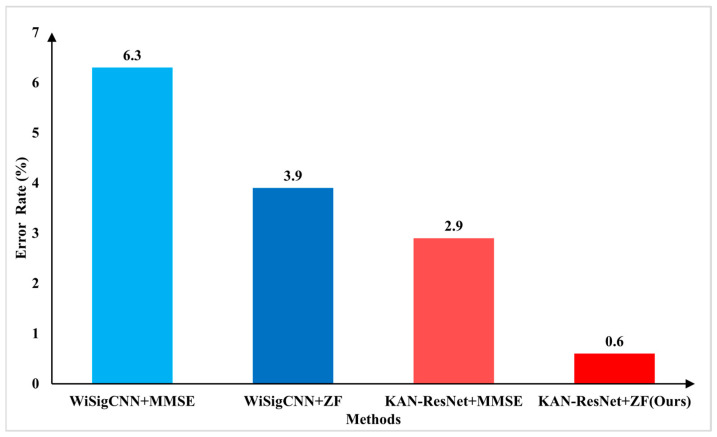
Error rate comparison between KAN-ResNet + ZF (Ours) and WiSigCNN + MMSE in the D1 scenario.

Figure 11 shows that, when testing on March 21st data in the D1 training scenario, the KAN-ResNet model achieved a lower error rate compared to the WiSigCNN when using the same equalization method. By observing Table 3, the training epochs, total training time, and average inference time are analyzed under different training scenarios. The results show that KAN-ResNet converges significantly faster than traditional models. Although its inference time is slightly longer, the difference is negligible in practical applications. Based on Table 2 and Table 4, KAN-ResNet replaces traditional fully connected layers with KAN modules. By using learnable activation functions, the model achieves better feature classification. In addition, the optimized regularization loss, as described in Section 4, further improves the robustness of the network. The model also reduces about 3078 parameters, helping maintain low memory usage, which is beneficial for deployment in resource-constrained environments. Figure 11 shows that KAN-ResNet combined with ZF equalization achieves an error rate of only 0.6%, while WiSigCNN combined with MMSE equalization registers an error rate of 6.3%. This demonstrates that the proposed approach has great potential and practical value in addressing the environmental and multipath effects encountered in RFFI applications. Comparing Table 2 and Table 4, it can be found that KAN-ResNet combined with ZF equalization outperforms the WiSigCNN combined with MMSE equalization in different training scenarios D1, D2, and D3 for different dates of test data.

**Figure 12:**
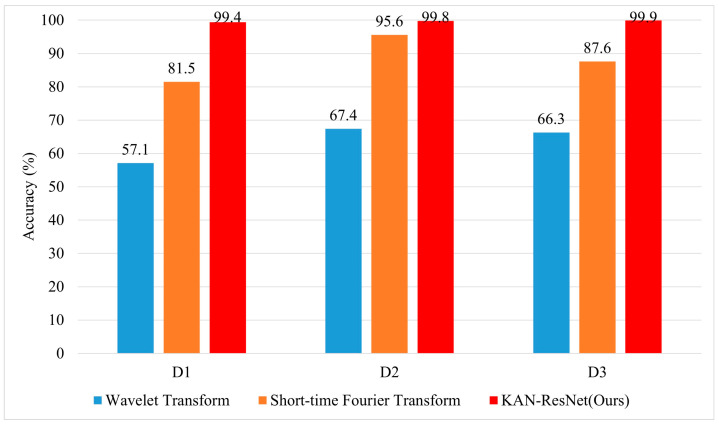
Comparison of classification accuracy based on expert features (STFT, Wavelet) with KAN-ResNet in the ManySig-ZF dataset D1, D2, D3 scenarios.

**Figure 13:**
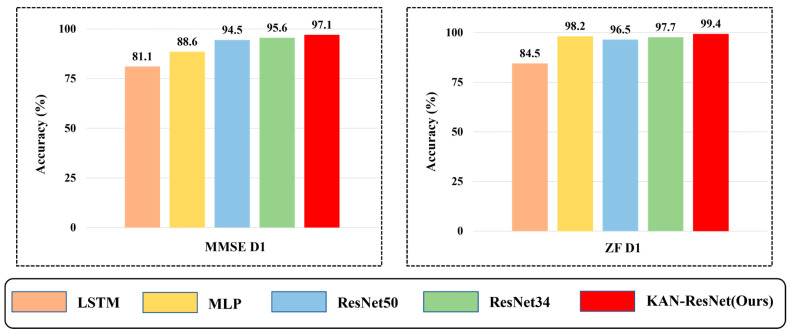
Comparison of experimental classification accuracy of different neural networks on MMSE and ZF equalization data.

To further highlight the superiority of the KAN-ResNet model in the RFFI task related to time-varying channels, we compare it with traditional expert feature extraction methods, including short-time Fourier transform (STFT) [48] and Wavelet Transform [49], as well as four algorithms: ResNet34, ResNet50, MLP [33], and LSTM [32]. The experimental results are presented in Figure 12 and Figure 13. As shown in Figure 12, Wavelet Transform achieves only 57.1% accuracy in the D1 scenario, while short-time Fourier transform reaches 81.5%. In contrast, KAN-ResNet achieves 99.4%. As the training data increase (D2, D3), the accuracy of Wavelet Transform and short-time Fourier transform improves but still lags behind KAN-ResNet. This comparison demonstrates that KAN-ResNet is more effective in mitigating environmental and multipath interference across different days, achieving higher recognition accuracy and robustness than traditional expert feature-based methods. Expert feature-based RFFI relies on domain knowledge and requires selecting appropriate parameters for specific problems. Converting I/Q data into expert features increases computational complexity. Additionally, expert features may be closely tied to specific protocols. As shown in Figure 13, the experimental results demonstrate that KAN-ResNet outperformed the other four neural networks in both MMSE equalization and ZF equalization datasets. Moreover, when ZF equalization replaced MMSE equalization in the D1 training scenario, the KAN-ResNet model achieved a testing accuracy of 99.4%, the highest recorded accuracy to date. Thanks to KAN’s powerful ability to learn complex functions, the KAN-ResNet model classifies the extracted features more accurately, thereby improving RFFI accuracy. This demonstrates that the RFFI scheme, which uses ZF equalization and KAN-ResNet, has significant potential for time-varying channel applications. It is an ideal replacement for traditional device verification methods.

In this work, our overarching strategy for addressing potential RFFI attacks (e.g., spoofing) is to maximize the accuracy of legitimate transmitter identification. By leveraging the KAN-ResNet architecture, we focus on ensuring that only registered, authorized devices are recognized by the authentication system, thereby reducing the opportunities for unauthorized or cloned transmitters to pass as legitimate. While more specialized defenses (such as replay attack detection or GAN-based spoofing analysis) may be explored in future research, our current results demonstrate that a high-fidelity, robust RFFI model provides a solid foundation for mitigating spoofing risks in real-world deployments.

## 7. Discussion

In this paper, we proposed a novel RFFI framework with ZF equalization and KAN-ResNet models. Specifically, ZF equalization effectively mitigates residual channel effects and multipath interference resulting from incomplete channel estimation and frequency offset, thereby enabling more precise RF fingerprint extraction and supporting robust device authentication in real-world wireless scenarios. However, in environments with significant multipath propagation, ZF equalization may also amplify noise when the channel matrix is nearly singular or when the signal-to-noise ratio is low. Furthermore, accurate channel estimation critically determines its performance; errors in estimation can lead to suboptimal equalization under severe multipath conditions.

Compared to traditional methods that rely on fixed activation functions and simple fully connected layers, the proposed KAN-ResNet model incorporates learnable B-spline basis functions into a residual architecture. This enables the network to adaptively capture the complex nonlinear features inherent in RFFs, enhancing feature recognition and robustness against time-varying channel effects. Combined with the KAN-ResNet model, this framework demonstrates significant advantages in feature extraction and device identification. In complex scenarios, the Wi-Fi transmitter identification accuracy reaches 99.4%, reducing the error rate from 6.3% to 0.6% compared to the WiSigCNN-MMSE RFFI framework. These outstanding experimental results confirm the effectiveness of the proposed method under the tested conditions.

Furthermore, combining ZF equalization with the KAN-ResNet architecture effectively overcomes challenges such as residual channel effects, multipath interference, and channel estimation errors inherent in traditional methods. This integration significantly enhances feature extraction, improving the accuracy and robustness of RF fingerprint identification systems. As a result, the proposed approach demonstrates high adaptability in real-world wireless networks with unpredictable channel conditions. Although controlled experiments show clear performance improvements, we acknowledge the limitations of ZF equalization in real scenarios with severe multipath effects. Future work may explore adaptive or hybrid equalization schemes to further enhance system robustness.

Moreover, the residual structure in the KAN-ResNet model facilitates efficient gradient propagation and iterative feature refinement. This capability is particularly important for capturing subtle hardware-induced variations in radio frequency signals, which traditional methods often struggle to achieve. By replacing conventional FC layers with KAN modules, the proposed model effectively controls the number of parameters, reducing the risk of overfitting while significantly enhancing generalization. This approach addresses some inherent challenges faced by deep learning-based RFFI systems and further demonstrates the performance advantages of the proposed method.

The comprehensive experimental results show that the proposed method maintains high accuracy and robustness under time-varying channel conditions, making it suitable for deployment in real world wireless networks with unpredictable channel environments. Integrating the KAN module into the ResNet architecture not only improves identification accuracy and computational efficiency but also enhances model interpretability, which is crucial for fault diagnosis and wireless security system optimization. Empirical evaluations on multiple Wi-Fi datasets demonstrate the adaptability and effectiveness of method across different tasks. These findings further highlight the potential of non-traditional network structures, such as KAN, and their significance in advancing the widespread adoption of RFFI technology.

For future work, we propose the following research directions:

1. Explore adaptive or hybrid equalization techniques based on real-time channel conditions to enable dynamic switching between ZF, MMSE, or their combinations;
2. Extend the KAN-ResNet framework to RF fingerprinting (see Appendix A for definition) of other wireless signals, such as Bluetooth, Zigbee, and LoRa, and evaluate its performance under time-varying channel conditions;
3. Develop efficient model compression and optimization strategies to further reduce inference time and memory usage, making deployment feasible on resource-constrained devices;
4. Investigate improved real-time channel estimation methods to enhance the overall system robustness.

Additionally, since wireless signal transmission is an interactive process, the receiver may be affected by factors such as aging, environmental changes, and hardware degradation over long-term operation. These factors can introduce potential interference to the RFFI system. Therefore, further research on the ability of this method to address these issues is an important direction for future work.

## Figures and Tables

**Table 1:** Experimental scenario setting.

Experiment		D1	D2	D3
Train		March 1st	March 1st, March 8th	March 1st, March 8th, March 21st
Test	Same Day	March 1st	March 1st, March 8th	March 1st, March 8th, March 21st
	Different Days	March 21st	March 21st	March 21st

**Table 2:** Experimental classification accuracy of MMSE-equalized data in different experimental scenarios.

Method	Test Scenario	D1 (%)	D2 (%)	D3 (%)
WiSigCNN	Same Day	100	100	100
	Different Days	93.7 ± 8.7	96.7 ± 3.1	99.2 ± 1.5
ResNet_FC	Same Day	100	100	100
	Different Days	94.6 ± 2.6	99.6 ± 0.7	99.7 ± 0.3
KAN-ResNet (Ours)	Same Day	100	100	100
	Different Days	97.6 ± 1.3	99.4 ± 0.9	99.7 ± 0.1

**Table 3:** Comparison of epochs and times between KAN-ResNet and CNN.

Network	Experimental Scenario	Epochs	Training Time (s)	Test Time (s)
WiSigCNN	D1	150	154	1.34
	D2	150	313	1.34
	D3	150	474	1.25
KAN-ResNet (Ours)	D1	12	46	1.75
	D2	13	98	1.75
	D3	12	133	2.21

**Table 4:** Testing accuracy of different networks on ZF-equalized data.

Method	Test Scenario	D1 (%)	D2 (%)	D3 (%)
WiSigCNN	Same Day	100	100	100
	Different Days	95.5 ± 2.4	99.1 ± 0.3	99.3 ± 1.1
ResNet_FC	Same Day	99.8	100	100
	Different Days	97.4 ± 0.2	99.4 ± 1.9	99.8 ± 0.0
KAN-ResNet (Ours)	Same Day	100	100	100
	Different Days	99.4 ± 0.1	99.8 ± 0.1	99.9 ± 0.1

**Table 5:** Testing precision of different networks on ZF-equalized data.

Method	Test Scenario	D1 (%)	D2 (%)	D3 (%)
WiSigCNN	Same Day	100	100	100
	Different Days	96.2	99.2	99.5
ResNet_FC	Same Day	99.8	100	100
	Different Days	97.6	99.8	99.8
KAN-ResNet (Ours)	Same Day	99.8	100	100
	Different Days	99.4	99.8	99.9

**Table 6:** Testing recall of different networks on ZF-equalized data.

Method	Test Scenario	D1 (%)	D2 (%)	D3 (%)
WiSigCNN	Same Day	100	100	100
	Different Days	96.1	99.1	99.5
ResNet_FC	Same Day	99.8	100	100
	Different Days	97.5	99.8	99.8
KAN-ResNet (Ours)	Same Day	99.8	100	100
	Different Days	99.4	99.8	99.9

**Table 7:** Testing F1-score of different networks on ZF-equalized data.

Method	Test Scenario	D1 (%)	D2 (%)	D3 (%)
WiSigCNN	Same Day	100	100	100
	Different Days	96.1	99.2	99.6
ResNet_FC	Same Day	99.8	100	100
	Different Days	97.5	99.8	99.8
KAN-ResNet (Ours)	Same Day	99.8	100	100
	Different Days	99.4	99.8	99.9

**Table 8:** Testing accuracy of various grid_eps values on ZF equalization data.

The Value of Grid_eps	Test Scenario	D1 (%)	D2 (%)	D3 (%)
0.01	Different Days	96.9	93.3	96.1
0.02		99.4	99.8	99.9
0.05		96.4	98.9	99.9
0.1		97.9	96.1	97.5

## Data Availability

The WiSig dataset used in this paper is available through the following link: (https://cores.ee.ucla.edu/downloads/datasets/wisig/#/downloads (accessed on 3 June 2024)).

## Abbreviations

The following abbreviations are used in this manuscript:

RFFI	Radio Frequency Fingerprint Identification
IOT	Internet of Things
MAC	Media Access Control
RFF	Radio Frequency Fingerprint
CNN	Convolutional Neural Network
ResNet	Residual Network
LSTM	Long Short-Term Memory network
MLP	Multilayer Perceptrons
CGAN	Conditional Generative Adversarial Network
JCAE-CNN	Joint Convolutional Auto-Encoder and Convolutional Neural Network
MMSE	Minimum Mean Square Error
ZF	Zero-Forcing
KAN	Kolmogorov–Arnold Network
ISI	Inter-Symbol Interference
FIR	Finite Impulse Response
LMS	Least Mean Square
FDE	Frequency-Domain Equalization
DAC	Digital-to-Analog Converter
LO	Local Oscillator
PA	Power Amplifier
LNA	Low Noise Amplifier
ADC	Analog-to-Digital Converter
EFO	Estimation of Frequency Offset
PLCP	Physical Layer Convergence Protocol
PPDU	PLCP Protocol Data Unit
BN	Batch Normalization
ReLU	Rectified Linear Unit
SiLU	Sigmoid Linear Unit
FC	Fully Connected
SNR	Signal-to-Noise Ratio
STFT	Short-Time Fourier Transform

## Appendix A

Glossary of terms:

- Fingerprinting: Refers to the process of extracting RF fingerprints, where unique “fingerprints” are formed by analyzing the inherent hardware characteristics present in a device’s transmitted signal;
- Identification: Involves recognizing a device based on its extracted RF fingerprint, determining its specific identity;
- Authentication: Uses RF fingerprints for security verification, ensuring that a device is legitimate and authentic, thereby enabling access control.
